# Global phylogenomic analyses of *Mycobacterium abscessus* provide context for non cystic fibrosis infections and the evolution of antibiotic resistance

**DOI:** 10.1038/s41467-021-25484-9

**Published:** 2021-08-26

**Authors:** Ryan A. Bronson, Chhavi Gupta, Abigail L. Manson, Jan A. Nguyen, Asli Bahadirli-Talbott, Nicole M. Parrish, Ashlee M. Earl, Keira A. Cohen

**Affiliations:** 1 Infectious Disease and Microbiome Program, Broad Institute of MIT and Harvard, Cambridge, MA US; 2 Division of Pulmonary and Critical Care Medicine, Johns Hopkins University School of Medicine, Baltimore, MD US; 3 Department of Pathology, Johns Hopkins University School of Medicine, Baltimore, MD US

**Keywords:** Phylogenetics, Phylogenomics, Antibiotics, Bacterial genomics

## Abstract

*Mycobacterium abscessus* (MAB) is an emerging pathogen that leads to chronic lung infections. To date, the global population structure of non-cystic fibrosis (CF) MAB and evolutionary patterns of drug resistance emergence have not been investigated. Here we construct a global dataset of 1,279 MAB whole genomes from CF or non-CF patients. We utilize whole genome analysis to assess relatedness, phylogeography, and drug resistance evolution. MAB isolates from CF and non-CF hosts are interspersed throughout the phylogeny, such that the majority of dominant circulating clones include isolates from both populations, indicating that global spread of MAB clones is not sequestered to CF contexts. We identify a large clade of *M. abscessus* harboring the *erm*(41) T28C mutation, predicted to confer macrolide susceptibility in this otherwise macrolide-resistant species. Identification of multiple evolutionary events within this clade, consistent with regain of wild type, intrinsic macrolide resistance, underscores the critical importance of macrolides in MAB.

## Abstract (web summary)

Mycobacterium abscessus is an emerging infection that usually affects patients with structural lung diseases such as cystic fibrosis (CF). Here, the authors use phylogenetic analyses to demonstrate close relationships between isolates from CF and non-CF patients and identify antibiotic resistance markers.

## Introduction

*Mycobacterium abscessus* (MAB) comprises emerging opportunistic pathogens of increasing clinical importance. Categorized as rapidly growing mycobacteria (RGM), MAB is includes three subspecies: *M. abscessus abscessus* (*M. abscessus*), *M. abscessus massiliense* (*M. massiliense*), and *M. abscessus bolletii* (*M. bolletii*)^1^. Chronic pulmonary infection with MAB occurs most commonly among individuals with structural lung diseases—such as cystic fibrosis (CF), noncystic fibrosis bronchiectasis or chronic obstructive pulmonary disease—or in immunocompromised hosts; however, infections can also occur in immunocompetent hosts without known risk factors^2^. Severe extrapulmonary disease, including surgical site infections, also occurs^3^. Clinical management of MAB presents unique challenges due to limited numbers of effective antibiotics, prolonged course lengths, and frequent treatment-related toxicities^4^. Treatment generally consists of multidrug antibiotic regimens that include an oral macrolide (azithromycin or clarithromycin) and intravenous amikacin (an aminoglycoside), in combination with intravenous β-lactams, such as imipenem or cefoxitin, and other drug classes such as tetracyclines, oxazolidinones, or quinolones^5–7^. Treatment outcomes are poor even with administration of prolonged multidrug regimens of 18 months and longer, and reinfection is common^8–10^.

As MAB have previously been isolated from the environment, acquisition of infection is thought to originate primarily from contaminated environmental sources^11,12^. However, recent genomic studies by Bryant et al. analyzing the global phylogeography of MAB in CF have identified multiple clusters of MAB isolates separated by few SNPs that were isolated from individuals in different countries, which have been termed dominant circulating clones^13,14^. The global scope and significance of MAB dominant circulating clones has not been fully characterized, and the mechanism by which mycobacterial isolates collected from CF patients from different parts of the globe could be separated by so few SNPs is unclear^13,14^. As the Cystic Fibrosis Foundation guidelines recommend that CF patients have quarterly clinic visits in a designated CF center^15^, these findings raised concern for potential for person-to-person nosocomial transmission among CF patients, which was further evaluated by epidemiological contact tracing paired with genomic analysis^13,14,16^.

Relative to CF, less is known about the population structure of MAB strains that infect non-CF hosts. Previous large-scale phylogeographic studies have included isolates from patients either with CF or without CF^13,14,17,18^, and studies that have performed genomic analyses on strains from both patient populations have been small scale^19–21^. Thus, there are limited global data regarding the population structure of MAB isolates across CF and non-CF contexts, including whether there are MAB groups that are specific for CF or non-CF hosts, or whether non-CF individuals participate in MAB transmission dynamics similar to those observed for CF hosts.

Despite MAB being labeled an “antibiotic nightmare”^22^, the relationship between clusters in the MAB phylogeography and drug resistance remains unexplored. MAB exhibits both acquired and innate antibiotic resistance, limiting antibiotic selection and contributing to poor treatment outcomes^22^. Acquired drug resistance to amikacin and macrolides is conferred by mutations in the ribosomal RNA genes *rrs* (16 S rRNA) and *rrl* (23 S rRNA), respectively^23–25^. In addition to acquired resistance, innate inducible resistance to macrolides in certain MAB subspecies is conferred by upregulation of the erythromycin ribosome methyltransferase (*erm*(41)) gene upon exposure to macrolides^26^. Subsequently, *erm*(41)-mediated methylation of the macrolide 23 S binding site results in steric inhibition of macrolide binding and a phenotype of inducible macrolide resistance. In contrast, isolates with a nonfunctional *erm*(41) gene—either due to a large deletion or a T28C substitution—do not exhibit inducible macrolide resistance^26–28^. Differential *erm*(41) genotypes observed among the MAB subspecies correlate with clinical outcomes^29–32^. For instance, *M. massiliense* isolates contain a truncated *erm*(41) gene owing to a large deletion^26–28^ and have been associated with improved clinical treatment outcomes, which is attributed to retained macrolide susceptibility in this subspecies. Despite knowledge of the resistance mechanisms for macrolides and aminoglycosides and the critical importance of these drugs for the successful treatment of the MAB, the evolutionary patterns of drug resistance emergence have not been explored.

We constructed a global, large-scale dataset of diverse clinical MAB isolate genomes to examine the phylogeographic relationships between isolates from both CF and non-CF patients, and to examine patterns of drug resistance evolution.

## Results

### Dominant circulating clones of *M. abscessus* encompass both CF and non-CF patient populations

While prior phylogenetic analyses identified predominant clones of *M. abscessus* and *M. massiliense* circulating among geographically separated CF patients (dominant circulating clones), the relationship between these clones and patients without CF (non-CF) has not been characterized on a global scale. In order to place non-CF patient isolates into this context, we constructed a large and geographically diverse dataset including 1279 clinical MAB isolate genome assemblies from 690 unique patients: 514 with and 176 without CF (Methods; Supplementary Table 1). This dataset included: (i) 1086 previously published assemblies representing 500 unique individuals with CF from seven countries in Europe, United States, and Australia, collected from 2000 to 2014^14^ (the Bryant sample set); (ii) 162 previously published assemblies representing 162 individuals without CF from 16 provinces in China, collected between 2012 and 2015^17^ (the Li sample set); (iii) 30 assemblies newly generated as part of this study using a combination of Illumina and Oxford Nanopore Technology (ONT) data, representing isolates from 27 individuals, 14 with and 13 without CF, collected in the United States between 2015 and 2017; and (iv) the reference strain *M. abscessus* ATCC 19977^33^, which was resequenced as part of this study. Together, approximately a quarter of these isolates (26% and 27% of *M. abscessus* and *M. massiliense*, respectively) were from patients without CF. All 30 *M. bolletii* isolates were from the Bryant dataset and represented CF patients with limited geographic diversity.

**Fig. 1:**
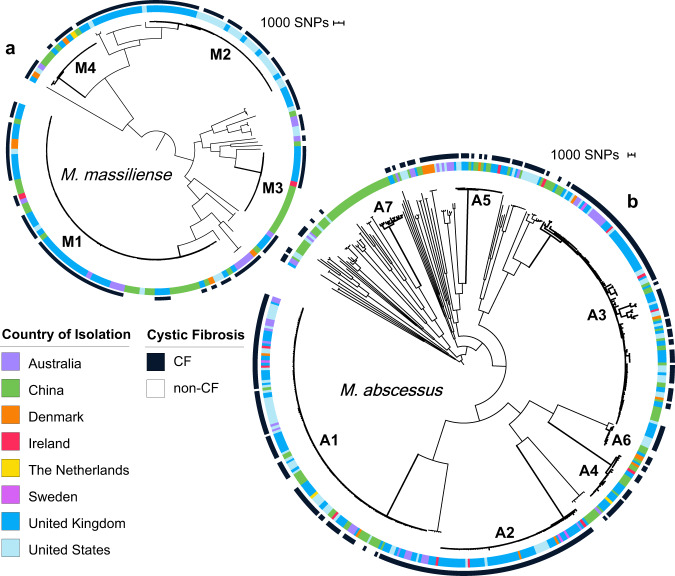
Isolates of different cystic fibrosis (CF) status and country of isolation are dispersed across MAB dominant circulating clones. Single-copy core phylogenetic trees for **a** *M. massiliense* and **b** *M. abscessus*, including one isolate per patient. Trees were constructed using RAxML^66^ after recombination removal. The inner ring indicates the country of isolation, and the outer ring indicates whether the sample originated from a person with a diagnosis of CF (black) or without CF (white). DCCs (Supplementary Table 3; Supplementary Fig. 1) with at least 10 isolates of *M. abscessus* (A1-A6) or *M. massiliense* (M1-M4) are numbered in descending order of size with bold branches. DCCs A1, A2, and M1 corresponded to *M. abscessus* clusters 1 and 2, and *M. massiliense* cluster 1, respectively, from Bryant et al. ^14^. The corresponding data for *M. bolletii* is shown in Supplementary Fig. 1a.

From these 1279 isolates, we compiled a set of 696 isolate assemblies, selected to represent a single isolate from each of 685 unique individuals, together with secondary isolates from individuals from whom more than one subspecies was isolated^13,14^. Using a phylogenetic analysis of this set anchored by known subspecies references, we identified 495 representatives from *M. abscessus*, 171 from *M. massiliense*, and 30 from *M. bolletii*. To better resolve relationships among isolates, we generated a core genome alignment for each subspecies that was cleaned of suspected recombined regions using Gubbins^34^, which we used to create high-resolution subspecies-specific phylogenies (Methods). As previously reported^14^, each subspecies tree was composed of long branches, indicating diversity among patient isolates, as well as regions of short branches, indicating recent ancestral origins for some (Fig. 1, Supplementary Fig. 1). When we overlaid CF status, we observed that isolates from both CF and non-CF hosts were broadly distributed across the *M. abscessus* and *M. massiliense* phylogenies and were separated by both long and short branches, indicating that many CF and non-CF isolates were closely related.

To determine whether non-CF isolates exhibited the same clonal trends previously reported in CF isolates^14^, we approximated the clusters identified in Bryant et al. using a core SNP threshold previously used to partition bacterial phylogenies into clades^35,36^. This SNP threshold captured the previously defined structure in each subspecies phylogeny^14^ (Supplementary Figs. 1 and 2; Methods), and were in broad agreement with those from Bryant et al. where the vast majority of patient isolates fell within clusters representing multiple treatment centers. We also observed transcontinental clusters, or dominant circulating clones (DCCs), that corresponded closely to those reported by Bryant et al. (A1, A2, M1, Fig. 1; Supplementary Figs. 1 and 2) as well as additional large transcontinental clusters of 10 or more isolates within *M. abscessus* (A3-A6) and *M. massiliense* (M2-M4) (Fig. 1) that emerged by bringing together data from different studies. Notably, each of these DCCs contained isolates from both CF and non-CF patients. In total, 73% of patients, including 77% of CF patients and 60% of non-CF patients, harbored isolates from these global DCCs (Supplementary Fig. 2; Supplementary Table 3), indicating that these strains are not restricted to a CF patient population. Higher resolution trees constructed for each DCC further confirmed nesting of CF and non-CF isolates (Supplementary Fig. 3).

### SNP distances revealed close relationships between epidemiologically unrelated patients of same, as well as different CF status

**Fig. 2:**
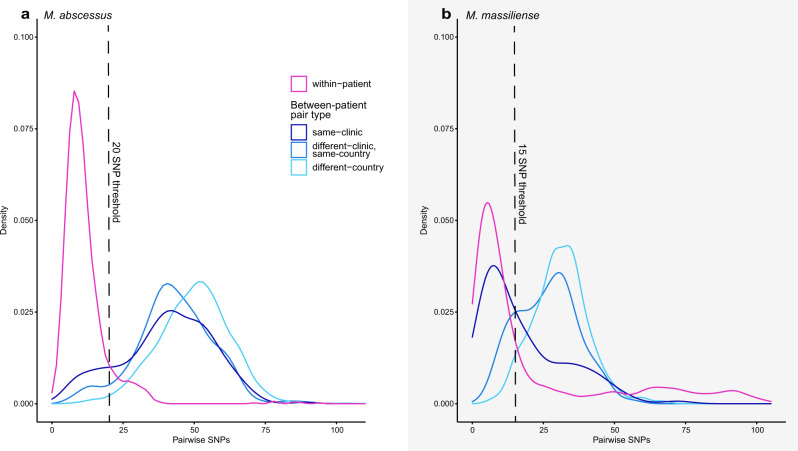
Pairwise SNP differences were significantly lower for isolates from the same patient than for those from different patients. Distributions of pairwise SNPs from within-patient and between-patient isolates in **a** *M. abscessus* and **b** *M. massiliense* were used to calculate a SNP threshold for closely related isolates (Supplementary Fig. 3; Methods). Pairwise combinations of isolates from the Li dataset were removed from this figure, as we did not have information on the specific clinic or province of origin for these isolates. Where data were available (for the Bryant dataset and the newly sequenced isolates), we also show the breakdown of between-patient isolates into same-clinic, different-clinic and same-country, and different-country pairs.

While the apparent close genetic relationships between certain CF patient isolates could be due to nosocomial transmission in CF care centers, non-CF MAB patients do not routinely receive centralized medical care for their infections; thus, non-CF MAB isolates are less likely to be epidemiologically related. To determine whether the close connections previously observed between some pairs of CF isolates could also be observed between CF and non-CF pairs, we calculated the pairwise genetic distance between isolates from the same circulating clone using whole-genome assemblies, after removing regions of suspected recombination (Methods). These pairwise comparisons took into account a larger fraction of the genome across pairs (88–100% of genes) than the core genome-based approach we used to create phylogenies (~50–75% of genes), and were not biased by the choice of a single reference, as is the case for comparative genomic studies that rely upon reference alignment^20^. To calibrate this analysis, we compared pairwise genetic distances between isolates cultured longitudinally from the same patient (within-patient pairs) to those cultured from unrelated patients (between-patient pairs). As expected, within-patient pairs were significantly more closely related than between-patient pairs (*p* < 2.2 × 10^−16^ for both *M. abscessus* and *M. massiliense*; two-sample Wilcoxon test). We also compared SNP distances separating isolates from different patients in the same clinic, from patients in different clinics in the same country, and from patients in different countries. As expected from findings by Bryant and colleagues, same-clinic pairs were separated by fewer SNPs on average than pairs unlikely to be epidemiologically related (*p* < 2.2 × 10^−16^ for both *M. abscessus* and *M. massiliense*; two-sample Wilcoxon test). Using the separation in the distribution of pairwise SNPs when comparing within- and between-patient pairs (Fig. 2), we calculated an optimized threshold for the number of SNPs that would best classify within-patient pairs, establishing a SNP threshold with which to identify very closely related isolates (Methods; Supplementary Fig. 4). The optimal threshold determined for each subspecies—20 SNPs for *M. abscessus* and 15 SNPs for *M. massiliense*—were similar to those used by Bryant et al., and correctly classified 94% and 72% of pairs as within- or between-patient, for the two subspecies, respectively. The classification accuracy was lower in *M. massiliense* due to the inclusion of isolates from within-clinic outbreaks, many of which were epidemiologically confirmed, in the Bryant dataset.

Applying these subspecies-specific thresholds to our global dataset, we found many (46) cases of very close relationships among pairs of isolates with no obvious epidemiological links (Supplementary Table 4), including 14 examples of pairs linking isolates from CF and non-CF patients being treated in different countries. For example, despite comparing >99% of genes, a CF and non-CF patient pair treated in the United Kingdom and China was separated by only 10 pairwise SNPs, suggesting that global circulation of highly related MAB isolates is not just occurring within the CF community.

### Modest levels of resistance to clarithromycin and amikacin predicted across our large multi-study dataset

Beyond refining phylogeography, gaining an improved understanding of MAB global drug resistance is of critical importance for this difficult to treat pathogen. Macrolides (clarithromycin or azithromycin) and amikacin are key components of guideline-based MAB multidrug regimens^5–7^ and the genotypic resistance mechanisms to these drugs are well established^23–26,36,37^. For these drugs we confirmed the phenotype/genotype relationship of a defined set of drug resistance mutations (Supplementary Table 5), using the 30 MAB clinical isolates that were newly sequenced for this study (see Supplementary Note 1).

**Fig. 3:**
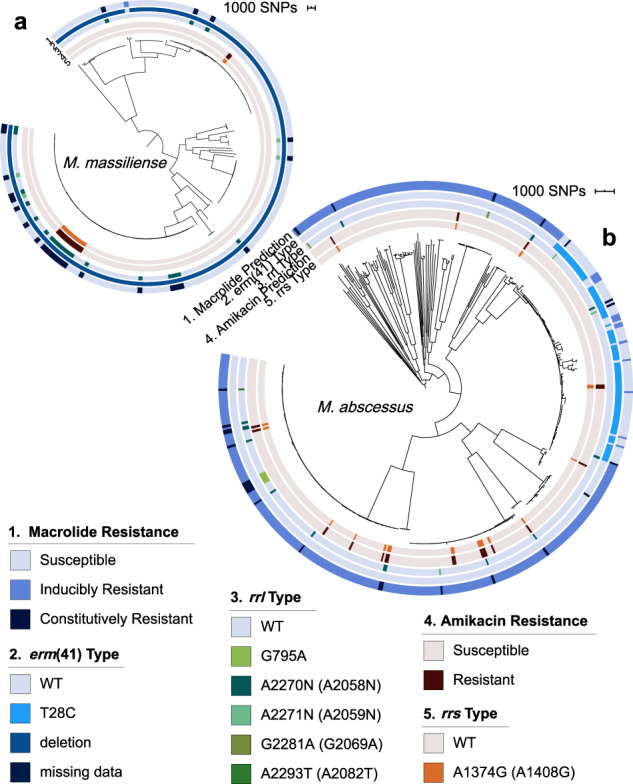
Predicted phenotypic resistance to clarithromycin and amikacin in MAB is subspecies specific. Predicted resistance in **a** *M. massiliense* and **b** *M. abscessus* to clarithromycin. From outside to inside, the rings represent: (1) predicted macrolide resistance, based on genotypic resistance markers in *erm*(41) and *rrl*. (2) erm(41) variants which confer susceptibility to clarithromycin. Wild-type *erm*(41) in the absence of *rrl* SNPs is associated with inducible clarithromycin resistance. The *erm*(41) T28C variant identified in clone A3 only, while other variants appear largely de novo throughout the subspecies, with the exception of potential expansion of resistance in DCCs A1, A3, and M1. (3) *rrl* variants which confer constitutive resistance to clarithromycin. (4) Predicted resistance to amikacin based on genotypic resistance markers in *rrs*. (5) *rrs* variant A1374G (*E. coli* A1408G), which confers resistance to amikacin.

As known *rrs, rrl*, and *erm*(41) drug resistance markers were 100% specific for observed resistance phenotypes for clarithromycin and amikacin in our set of 30 phenotyped isolates (Supplementary Fig. 5), we sought to gauge the fraction of isolates across our broader, geographically dispersed set of samples that were resistant to these two drugs (Fig. 3). After filtering to include only a single isolate per patient, we used the same dataset of 696 total MAB isolates to predict constitutive macrolide resistance, inducible macrolide resistance, and amikacin resistance, which was found in 7.3%, 58.9%, and 4.3% of isolates, respectively (Table 1; Fig. 3).

**Table 1:** Antimicrobial resistance markers for macrolides by MAB subspecies.

Subspecies	*erm*(41) variant^a^	*rrl* resistance variant^a,b^	Macrolide resistance phenotype	Fraction of *erm* variant (%)
*M. abscessus*	None	Absent	Inducible resistance	378/399 (94.7%)
		Present	Constitutive resistance	21/399 (5.3%)
	T28C^c^	Absent	Susceptible	91/95 (95.8%)
		Present	Constitutive resistance	4/95 (4.2%)
*M. massiliense*	None	Absent	Inducible resistance	1/1 (100.0%)
	Large deletion^d^	Absent	Susceptible	144/170 (84.7%)
		Present	Constitutive resistance	26/170 (15.3%)
*M. bolletii*	None	Present	Inducible resistance	30/30 (100.0%)

^a^Macrolide resistance is dependent on *erm*(41) and *rrl* (23 S rRNA), and most MAB strains are innately inducibly resistant to macrolides (most of *M. abscessus*, nearly no *M. massiliense*, all of *M. bolletii*). Functional *erm*(41) confers inducible resistance, but previously characterized *erm*(41) variants were associated with susceptibility to macrolides.

^b^SNPs in rrl are associated with constitutive resistance regardless of *erm*(41) background. For *rrl* resistance variants and specific SNP frequencies, see Supplementary Tables 5 and 7.

^c^The T28C variant confers susceptibility, and was only present in *M. abscessus*.

^d^A large deletion (spanning position 28) in *erm*(41) is known to persist in *M. massiliense*, rendering nearly the whole subspecies susceptible to macrolides (all but one *M. massiliense* isolate, potentially due to recombination or mixed isolation).

Constitutive macrolide resistance, conferred by *rrl* mutations, was predicted in a total of 51 MAB isolates: 5.1% (25/495) of *M. abscessus* and 15.2% (26/171) of *M. massiliense* (Fig. 3). The most commonly identified *rrl* mutations were the canonical A2270N (*E. coli* A2058N) (35/51, 68.6%) and A2271N (*E. coli* A2059N) (8/51, 15.6%). Additional *rrl* mutations that are less well characterized (G795A, G2281A [*E. coli* A2069A], and A2293T [*E. coli* A2082C]) but associated with macrolide resistance^37,38^ were identified only in *M. abscessus*. In this large dataset, we did not identify any MAB isolates with *rrl* variants T371C, A1932G, or A2269G (*E. coli* A2057G), which have been previously reported in this species^37,38^.

Of the 470 *M. abscessus* isolates that lacked relevant *rrl* mutations, 76.6% (360) contained the wild-type *erm*(41) T28, and were thus predicted to exhibit inducible macrolide resistance. Another 95 (19.2%) contained *erm*(41) T28C^26,27^, and were predicted to be macrolide susceptible (Fig. 3; Table 1). All but one isolate of *M. massiliense* within this global dataset were confirmed to contain the canonical large *erm*(41) deletion^26–28^ that renders *erm*(41) nonfunctional. Thus, we predicted inducible resistance in only this single *M. massiliense* isolate with wild-type *erm*(41). However, it is possible that this isolate from the Bryant dataset^14^ represents an example of a mixed infection or contains contamination from a *M. abscessus* strain containing the wild-type *erm*(41), as we saw evidence of mixed infection among our newly sequenced isolates, as well as in several isolates from the Bryant et al. dataset (Methods). All 30 of the *M. bolletii* contained wild-type *erm*(41) with T28, indicative of inducible macrolide resistance.

For amikacin, rates of predicted resistance were low across all three subspecies (4.0%, 5.3%, and 3.3% for *M. abscessus*, *M. massiliense* and *M. bolletii*, respectively). The only marker associated with amikacin resistance that we identified was the canonical *rrs* A1374G mutation (*E. coli* A1408G)^24^. We searched for additional SNPs in this region of 16 S rRNA that had been identified in amikacin resistant strains through in vitro selection, including T1406A, C1409T (*E. coli* numbering)^39^; however, these were not present in this large global dataset suggesting that they may not be relevant in vivo.

Neither predicted macrolide resistance phenotypes nor their associated genotypes (variants of *rrl* and *erm*(41)) were significantly enriched in either patient type (Supplementary Table 6). Variants of *rrs*, and thus predicted resistance to amikacin, were significantly enriched in isolates from CF patients relative to non-CF. However, these resistant isolates were mostly epidemiologically-linked *M. massiliense* isolates, from a within-clinic outbreak in the Bryant dataset^14^, and thus unlikely to represent a broader trend.

### *M. abscessuserm*(41) T28C arose from a common ancestor, with convergent de novo reversion to wild-type T28

**Fig. 4:**
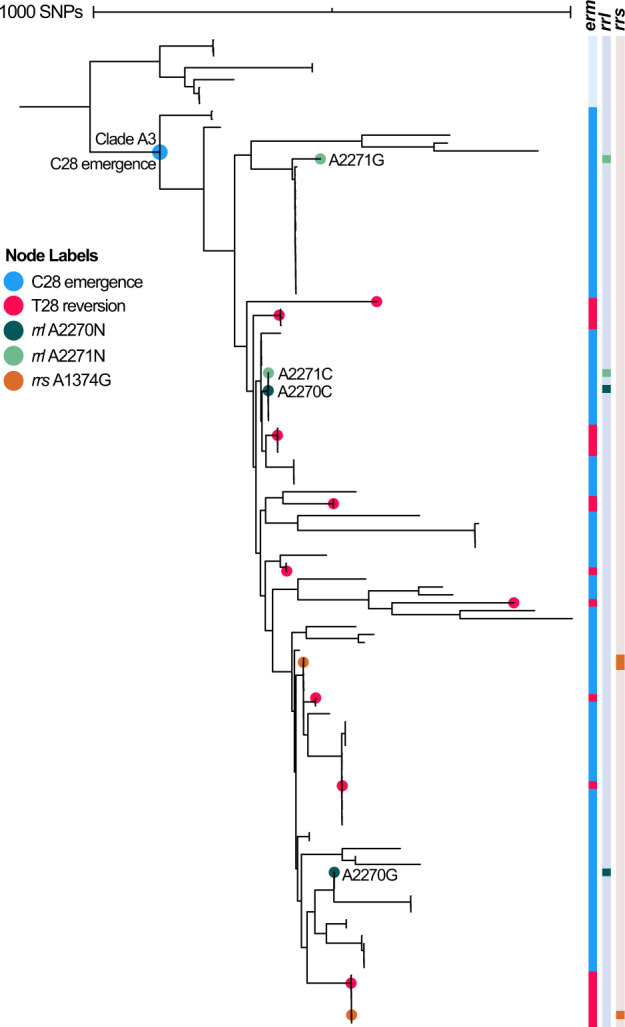
Molecular evolution of macrolide and amikacin susceptibility within *M. abscessus* clone A3. Midpoint rooted maximum-likelihood phylogeny of 116 members of clone A3. Each evolutionary gain or loss of a drug resistance mutation was assigned to its position on the phylogeny using parsimony. Given its basilar position on the phylogeny, the T28C *erm*(41) variant (blue circle), which precludes inducible macrolide resistance, occurred earlier in time, with subsequent-independent reversions back to T28 (red circles), indicating a regain of the inducible macrolide resistance phenotype. Within the clade, additional acquisitions of constitutive macrolide and amikacin resistance were also observed, with emergence of *rrl* A2270C (*E. coli* A2058C), *rrl* A2270G (*E. coli* A2058G), *rrl* A2271C (*E. coli* A2059C), *rrl* A2271G (*E. coli* A2059G), and *rrs* A1374G (*E. coli* A1408G). Bootstrap values (≥80%) support the independent, convergent acquisition of each instance of macrolide and amikacin resistance.

Although the majority of isolates in *M. abscessus* were predicted to have inducible or constitutive resistance to clarithromycin, there were 95 (19%) isolates within this subspecies in which an *erm*(41) T28C was identified, which, in the absence of *rrl* mutations, predicts susceptibility to clarithromycin. Of note, isolates with a T28C variant were identified to be present only within a single 116-member clade of *M. abscessus* (clone A3) (Fig. 4), and T28C mutations did not appear in any other MAB subspecies or within another clade of *M. abscessus*. Within clone A3, 82% of clade members contained the T28C and the remaining 21 isolates had a wild-type T28, which were distributed across the clade. The population structure of clone A3 suggests that the T28C *erm*(41) variant originated from a single ancestral event, and that identification of wild-type T28 within the clade is likely due to convergent de novo reversion that occurred independently at least nine times. Of note, there were four examples within clone A3 of de novo evolution of *rrl* mutations that confer constitutive resistance to macrolides, which supports selective drug pressure within the clade.

## Discussion

In a large global dataset constructed to look at the phylogeographic relationships between isolates from CF and non-CF patients, we concluded that the global spread of MAB clones is not sequestered within CF contexts. MAB isolates from patients both with and without CF were distributed across the phylogeny, nested within dominant circulating clones, and had surprisingly close relationships despite having no known epidemiological link. Furthermore, using genotypic markers across our global dataset to predict resistance to macrolides and amikacin—key drugs for treatment of MAB—we identified subspecies-specific resistance to macrolides, and modest levels of amikacin resistance in clinical isolates of MAB. In tracing the molecular evolution of drug resistance within MAB, we identified multiple evolutionary events consistent with regain of intrinsic macrolide resistance in an otherwise macrolide susceptible clade.

As previous large phylogenetic analyses of MAB were performed using only CF, or only non-CF patient isolates, it was unknown how MAB from these different patient populations were related. Combining isolates from patients with and without CF, we found that isolates from both patient populations exhibited similar phylogenetic patterns and close connections that could not be readily explained. In some cases, pairwise SNP distances between CF and non-CF patient isolates were extremely small (as few as 10 SNPs) and similar to distances observed between isolates from longitudinal samples within the same patient, suggesting that these cases derived from a recent common ancestor. In emerging literature conducted in parallel with this study, Lipworth and colleagues used an approach leveraging alignments to a single reference genome to identify clonal relationships between MAB isolates from CF and non-CF patients from England^40^. Patient metadata showed that direct transmission was unlikely to have explained the majority of close isolate relationships. While the high degree of relatedness reported in our study may indicate potential recent transmission, there was no obvious epidemiological link between isolates in these pairs, which included examples from different continents. Though travel and migration could account for some global transmission of clonal isolates, they are unlikely connections that would not explain the degree of widespread global dissemination of clonal strains we observed in the phylogenies. Therefore, we assumed that recent transmission would not explain close relationships between isolates from different countries or from patients with different CF status^20^. It has been previously hypothesized that recently emerged clones have spread locally via fomites and long-lived, infectious aerosols^14^, although the mechanism of global dispersion is unknown. For another member of this genus, *Mycobacterium chimaera*, it has been shown that medical heater-cooler devices manufactured at a common location resulted in the global dispersion of highly related isolates^41^. Our findings from a dataset combining isolates from CF and non-CF patients suggest that current guidelines that focus on MAB infection control efforts only in the context of CF patients cared for at CF centers^5,42^ should be reconsidered, as our results indicate more complex transmission dynamics. The relative distribution of CF and non-CF associated MAB isolates and the fact that many areas of the globe were not represented in our dataset of MAB genomes reflect the current state of the field. Our addition of new near-complete genomes to the current set of publicly available genomes will empower future investigation of genome dynamics in greater detail.

The phenomenon of isolates from CF and non-CF patients being intermingled in DCCs can be exploited for antimycobacterial drug development purposes: given that all but one of the DCCs contained isolates from both CF and non-CF patients, drug development need not be specific for each patient population. Isolates from each of the DCCs should be included in screens against novel compounds. The ATCC *M. abscessus* reference strain, 19977, which is often employed for drug discovery screening^43,44^, is a member of clone A1, the largest known DCC. Isolates from other clones also warrant inclusion in such drug discovery efforts in order to ensure that novel drugs have broad clinical utility. Furthermore, since initial studies have shown that isolates from DCCs are better able to survive within macrophages, cause more virulent infections in mice, and are associated with worse clinical outcomes^14^, their identification and use in drug discovery efforts seems particularly prudent. Ultimately, the clinical significance of MAB infection with distinct dominant circulating clones in CF and non-CF patients warrants further investigation.

Beyond rampant global transmission, MAB has been deemed a “new antibiotic nightmare”^22^ due to innate and acquired drug resistance, which complicates treatment. Multiple cellular mechanisms contribute to macrolide resistance in MAB, including innate inducible resistance in the setting of wild-type erythromycin ribosome methyltransferase *erm*(41), and acquired resistance conferred by mutations in 23 S rRNA (*rrl*). As the vast majority of observed macrolide resistance among our 30 newly sequenced MAB isolates was explained by known drug resistance markers, we used genotypic markers^37,38^ to infer drug susceptibility in our global dataset for which phenotypic DST was not available. Of note, genotypic resistance markers used in this study were specific, but not 100% sensitive, thus our quantification of drug resistance was likely conservative, and may underestimate resistance. Within our global dataset, identification of *rrl* mutations conferring acquired macrolide resistance occurred in 5.1% and 15.2% of *M. abscessus* and *M. massiliense* isolates, respectively. The higher frequency of constitutive resistance variants within *M. massiliens*e in comparison to *M. abscessus* may be a function of inclusion biases of the study population*—*for example, differential macrolide exposure between these two groups*—*but could also be indicative of heightened evolutionary pressure toward development of *rrl* mutations in an *M. massiliense* background due to presence of a nonfunctional *erm*(41) that lacks the inducible resistance phenotype.

The majority of the remaining *M. abscessus* isolates lacked *rrl* mutations and had a wild-type *erm*(41), indicative of an inducible macrolide-resistant phenotype, whereas 19.2% of *M. abscessus* contained *erm*(41) T28C, and were predicted to be macrolide susceptible. All but one isolate of *M. massiliense* within this global dataset were confirmed to contain the canonical large *erm*(41) deletion^26–28^ that renders *erm*(41) nonfunctional, and the deletion was not present in other subspecies, indicating that presence of this deletion confidently discriminates this subspecies. While the molecular mechanisms of resistance to macrolides and amikacin in MAB are well established, the genomic evolutionary patterns of drug resistance emergence within MAB were previously unexplored. The *erm*(41) T28C variant in *M. abscessus* has been previously associated with susceptibility to macrolides^26,27^; however, the phylogenetic relatedness of isolates containing this mutation had not been established. Our genomic analysis revealed that the T28C variant occurs in a single dominant circulating clone of *M. abscessus* (A3), suggesting emergence of this mutation occurred via a single evolutionary event rather than repeatedly across the phylogeny. This 116-member clade containing the T28C variant included isolates from both CF and non-CF individuals, and isolates from seven countries. Within this DCC, we observed multiple independent instances of suspected reversion back to the wild-type genotype T28, which is predicted to regain the phenotype of inducible macrolide resistance. Based on our parsimony-based analysis, the T28 reversions in these 21 isolates of clone A3 represent a minimum of nine distinct evolutionary events; however, this may be an underestimate of the true number of reversions due to possible convergent evolution which could obscure our ability to capture independent reversion events. In addition to T28 reversions, we observed four independent evolutionary gains of *rrl* mutations within clone A3 that confer constitutive, high-level macrolide resistance. Together, these phenomena suggest evolutionary pressure for regaining both inducible and intrinsic macrolide resistance within MAB.

With respect to amikacin resistance, acquired mutations in 16 S (*rrs*) explained the vast majority of phenotypic resistance to amikacin among our newly phenotyped isolates. There was a single *M. abscessus* isolate with unexplained phenotypic resistance to amikacin, which may be due to a novel resistance mechanism. In our global dataset, 4% (20/495) of *M. abscessus* and 5.3% (9/171) of *M. massiliense* genomes contained the canonical *rrs* A1374G (*E. coli* A1408G)^24^ that confers high-level phenotypic resistance. While additional *rrs* mutations that confer amikacin resistance in MAB have been identified through in vitro selection of drug resistance, none of these variants were present within the genomes of MAB clinical isolates. This discrepancy between resistance mutations that may exist in vitro, but are not found within clinical isolates, suggests that emergence of these mutations is rare or may carry an additional fitness cost that makes these untenable in vivo, as has been seen for other pathogens, including *M. tuberculosis*^45^. Similarly, caution should be taken when including laboratory-derived mutations in the design of molecular diagnostic tests for drug resistance in MAB.

Through whole-genome analysis, we identified that global clusters of MAB contain highly genetically similar isolates from both CF and non-CF hosts. Thus, infection control measures that typically focus on preventing MAB transmission only in CF centers should be revisited. In a molecular analysis of drug resistance evolution in MAB, we identified frequent evolutionary events of both the emergence of or reversion toward mutations that confer higher levels of macrolide resistance, which suggests differences in fitness or selective pressure either during the course of treatment or from the environment. Future genomic studies that include drug treatment history and clinical outcomes will be needed to determine the relationship between drug treatment and subsequent resistance in MAB and the clinical relevance of infection with a DCC. Additionally, the introduction of global environmental sampling from different mycobacterial reservoirs and sources will provide much needed context for the global dissemination of these clones. Improved understanding of transmission dynamics*—*including the degree of genomic diversity among environmental isolates*—*as well as the molecular determinants and risk factors for drug resistance will be necessary to combat this “antibiotic nightmare” of a human pathogen.

## Methods

### Specimen collection and drug susceptibility testing

Ethical approval for this study was granted by the Johns Hopkins University School of Medicine IRB (IRB00117772). The requirement for informed consent for specimen collection was waived by the IRB. Out of the approximately 150 MAB clinical isolates that were isolated in the Johns Hopkins Clinical Mycobacteriology Laboratory from 2015 to 2017, we selected 34 for inclusion in this study. Isolates were first identified by distinct host features to ensure representation from individuals both with and without cystic fibrosis. Inclusion was limited to only a single isolate per patient, rather than serial isolates, unless the serial isolates displayed differing drug susceptibility patterns. Isolates of diverse drug susceptibility patterns from both CF and non-CF hosts were randomly selected for inclusion. Single colonies of *M. abscessus* were picked from LJ slants, inoculated into 7H9 supplemented with Tween-80 (0.05%) and OADC (10%) and placed at 37 °C on a shaking incubator.

Drug susceptibility testing (DST) was performed prospectively in parallel to genomic DNA extraction (as described below). MIC determination was performed by broth microdilution using Sensititre RAPMYCO plates to a standard panel of 13 antibiotics that included clarithromycin and amikacin (Trek Diagnostic Systems/Biocentric, USA). Visible pellet growth was evaluated after 3 days of incubation at 30 °C for all drugs, and the lowest concentration of drug that did not show visible growth was recorded as the MIC to the respective drug. Plates were reincubated at 30 °C until 14 day of incubation to assess for inducible clarithromycin resistance. All MIC determination assays were performed in duplicate, and repeated a third time if there was significant variation in observed phenotype (ex: an MIC differed by more than one dilution).

### Whole-genome sequencing and assembly

We newly sequenced 34 isolates from Johns Hopkins Clinical Mycobacteriology Laboratory for this study using a combination of Illumina and Oxford Nanopore sequencing technologies. Genomic DNA was extracted from cultures grown in 7H9 using a modified CTAB-lysozyme method that included an additional phenol:chloroform purification. Standard inputs were used for the Illumina Nextera FLEX (Illumina, Inc., San Diego, CA). Library construction protocol following the manufacturer’s recommendations. Each library was uniquely dual indexed and assessed for concentration and size by Qubit (ThermoFisher Scientific, Waltham, MA) and TapeStation (Agilent Technologies, Santa Clara, CA), respectively. All libraries were pooled together and loaded onto a HiSeqX Illumina sequencer for 300 cycles to achieve 1.5 G of data per sample, as 150 base pair (bp), paired end read sets. The resulting sequencing data were of high quality and complexity and provided the intended depth of sequencing coverage to enable planned downstream analyses.

In addition, 600 nanograms of DNA from each sample were used as input into the Oxford Nanopore 1D ligation library construction protocol following the manufacturer’s recommendation. Samples were barcoded using the Native Barcoding Expansion 1–12 kit to run in batches of six samples per flow cell on a GridIon sequencer (Oxford Nanopore Technologies Ltd, Science Park, UK). Oxford Nanopore (ONT) reads were demultiplexed using Deepbinner (v0.2.0)^46^, trimmed any remaining adapter using Porechop (v0.2.3)^47^, and subsampled to ~50× depth of genome coverage. Illumina reads were trimmed of adapter using Trim Galore (v0.5.0)^48^ and subsampled to ~100× depth of genome coverage. Two Unicycler (v0.4.4, with default settings)^49^ hybrid assemblies were generated for each sample, one assembly combining the Illumina 100× dataset with the 50× subsampled ONT dataset and another assembly combining the Illumina 100× dataset with the full set of ONT reads (if >50×).

ONT reads were aligned to Unicycler contigs using minimap2 (v2.15)^50^. Illumina reads were aligned to Unicycler contigs using bwa mem (v0.7.17)^49,51^, and the resulting alignments were input to Pilon (v1.23)^52^ for assembly polishing. Contigs were screened for adapter sequence and then input to GAEMR (https://github.com/broadinstitute/GAEMR), which produced chart and metric tables for use in manual assembly analysis process. One assembly was corrected to include a small region of genome sequence missing in the original Unicycler assembly. Reads were submitted to SRA under BioProject PRJNA 523365.

We applied strict quality filters to exclude low quality and potentially mixed or contaminated samples, including removing isolates with (i) 16 S rRNA gene sequences from multiple species, identified using RNAmmer; (ii) genome assembly length longer than 50% higher than the average mycobacterial assembly length; (iii) substantial alignment to isolates from a different species, using GAEMR; (iv) unusually high phage content. Using these filters, we removed four genomes from downstream analysis.

### Dataset for comparative genomics

In constructing a dataset of comparator isolates, we brought together the largest global *M. abscessus* dataset from CF patients^14^, and unique global dataset containing isolates from non-CF patients where patient CF status and country of isolation were apparent^17^. Of our newly sequenced isolates, we selected a total of 27 (one per individual) to combine with (i) 509 isolates from Bryant et al. ^14^ representing 498 individual CF patients from seven countries: the United Kingdom, the United States, Australia, Sweden, Ireland, Denmark, and the Netherlands, sequenced with Illumina technology, and de novo assembled using Velvet^53^, and (ii) 160 assembled genomes of isolates from Shanghai Pulmonary Hospital^17^, each representing a unique non-CF patient and sequenced using Illumina technology and assembled using SPAdes^54^ in conjunction with BayesHammer^55^ (Supplementary Table 1).

After combining assemblies from all sources, we excluded three assemblies from the Li et al. ^17^ study that contained (i) likely species misidentification, (ii) >100 contigs, or (iii) evidence of contamination from a different species of nontuberculous mycobacteria. Although we saw no clear evidence for contamination from other MAB strains, our filters were not able to exclude contamination from closely related strains. However, our final dataset did not contain unexpectedly large assemblies, which would be indicative of the presence of multiple strains. We included 1086 assembled genomes from 500 patients in our comparative study, representing eight countries on four continents. One isolate per subspecies per patient was selected for our phylogenetic analyses, resulting in subspecies phylogenies with a total of 695 isolates from 684 unique patients (including 523 CF and 172 non-CF), plus the ATCC 19977 reference, which was isolated from a non-CF patient.

### Genome annotations

All 1279 genomes in our comparative analysis were uniformly reannotated using the Broad Institute’s prokaryotic annotation pipeline^56^. Protein-coding genes were predicted with Prodigal^57^ and filtered to remove genes with ≥70% overlap with tRNAs or rRNAs. tRNAs were identified by tRNAscan-SE^58^, and rRNA genes were predicted using RNAmmer^59^. Gene product names were assigned based on top BLAST hits against the SwissProt protein database (≥70% identity and ≥70% query coverage). Additional annotation analyses performed include Pfam^60^, TIGRfam^61^, KEGG^62^, and Enzyme Commission numbers.

### Generation of subspecies-specific core genome phylogenies

In order to categorize assemblies into subspecies prior to orthogroup clustering, we used a kmer similarity score to identify the subspecies reference closest to each isolate. We used high-quality assembled genomes from NCBI (see Supplementary Table 8) as references. Using this method, we identified 495 *M. abscessus* isolates, 171 *M. massiliense* isolates, and 30 *M. bolletii* isolates (Supplementary Table 1). We performed ortholog clustering separately for the set of isolates from unique patients for each subspecies set using SynerClust^63^.

In order to remove the effects of recombination and generate phylogenies, we created ordered multiple core genome alignments using parsnp from the HarvestTools Suite^64^. Subspecies-specific references used for alignments were (i) *M. abscessus* 19977 (our newly resequenced version, containing four substitutions compared to the NCBI assembly); (ii) *M. massiliense* 12082_5_84; and (iii) *M. bolletii* 10665_2_75. Core genomes comprised 51%, 81%, and 79% of the whole genomes for *M. abscessus*, *M. massiliense*, and *M. bolletii*, respectively. We used the HarvestTools-based alignment to predict and remove potential recombination using Gubbins^34^, which resulted in a recombination-removed core of 50%, 79%, and 75% of the genome for each subspecies, respectively. Gubbins generated phylogenetic trees for subspecies and DCCs using default RAxML-Light^65^ settings (GTRCAT). We constructed the tree in Fig. 3 using RAxML v7.3.3^66^ with 1000 starting trees, a parsimony input seed of 78960, and the GTRCAT model with random bootstrapping seed 12345. There was no collapsing performed as bootstrap supports for clades of interest was strong.

### Identification of clades

We used distance-based hierarchical clustering to identify closely related clades in MAB. We considered isolates to be clustered if joined by an internal node for which all branch lengths to leaves were less than or equal to 500 core SNPs. This was performed by using a 500 SNP threshold for hierarchical clustering, based on the core SNP distance matrix for each subspecies, using the hierarchical clustering toolkit from SciPy^67^. Dominant circulating clones were identified as clusters containing at least ten isolates from at least two countries.

### Calculation of pairwise SNP distances between assemblies

In order to gain a higher level of resolution, we tabulated the number of SNPs between all orthologs for each pair of assemblies, using all ortholog pairs identified by SynerClust for the pair of genomes. We identified and removed likely recombined regions by locating clusters of SNPs within sliding windows of size 1000–50,000 bp across the genome. As changing the window size did not significantly change the number of pairwise SNPs marked as recombined, we selected a 30 kb window size, corresponding to the length of recombination regions identified using ClonalFrameML by Tan et al. (2–34 kb)^68,69^, which to our knowledge is the only publication which assesses the size of recombined regions in M. abscessus. Any 30 kb window that included orthologs with more than one SNP within an ortholog was considered likely recombined and removed from the analysis.

### Optimizing pairwise SNP thresholds

We calculated precision and recall to assess how well different thresholds (between 0 and 100 SNPs) distinguished within-patient isolate pairs from between-patient isolate pairs. For each threshold, within-patient pairs that were separated by this number of SNPs or fewer were identified as “within-patient” and considered true positives; between-patient pairs separated by more SNPs than this threshold were identified as “between-patient” and considered true negatives. To optimize our threshold choice, we selected the threshold for each subspecies that had the highest F1 score. Because this analysis was independent of a reference genome, and because we optimized thresholds on a subspecies level, the thresholds established were higher resolution and less biased than those from previous studies based on reference genomes^20^.

### Prediction of drug resistance determinants for amikacin and clarithromycin

We predicted resistance genes for amikacin (mutations in the *rrs* 16 S rRNA gene) and clarithromycin (mutations in the *rrl* 23 S rRNA gene, as well as in *erm*(41)). We used RNAmmer to identify 16 S and 23 S rRNA sequences in each genome. *rrs* and *rrl* sequences were aligned to *E. coli* and mycobacterial references using the SINA tool^70^ from Silva (reference strains include those from the SINA tool database). We used these alignments to determine sequence coordinates with respect to known drug resistance SNPs in MAB. Eight assemblies from the Li and Bryant studies contained multiple copies of *rrs* and/or *rrl*; however, by alignment to close reference rRNA genes (chosen by SINA tool), we were able to identify which copies were MAB and which copies were likely due to contamination or mixed infection. In each of the eight assemblies of concern, there was a clear MAB rRNA gene accompanied by a non-MAB gene. We performed downstream analysis using the MAB rRNA genes only.

To identify *erm*(41) mutations, all MAB genomes were searched for the *erm*(41) gene using BLAST. *erm*(41) was present in all isolates, and an *erm*(41) alignment was produced using Muscle v3.8.31^71^.

Fisher’s exact test was employed to compare the relative frequencies of genotypic and phenotypic drug resistance between CF and non-CF host populations.

### Reporting summary

Further information on research design is available in the Nature Research Reporting Summary linked to this article.

## Supplementary information

Supplementary Information

Reporting Summary

## Data Availability

The genomic data generated in this study have been deposited in NCBI SRA under Bioproject PRJNA523365. The accession codes and phenotypic data generated in this study are provided in the Supplementary Table 2. Publicly available genome sequences utilized in this project include NCBI under BioProject PRJNA398137 and the European Nucleotide Archive under project accession PRJEB2779. GenBank assembly references used for MAB subspecies identification are found in Supplementary Table 8.
