# Supplementary material for: Global phylogenomic analyses of Mycobacterium abscessus provide context for non cystic fibrosis infections and the evolution of antibiotic resistance
Source: Nat Commun. 2021 Aug 26;12:5145. doi: 10.1038/s41467-021-25484-9 (PMC8390669; doi:10.1038/s41467-021-25484-9)
Supplement: Supplementary file 1 — Supplementary Information [file 41467_2021_25484_MOESM1_ESM.pdf]

Supplementary Information for:

Title: Global phylogenomic analyses of *Mycobacterium abscessus* provide context for non cystic fibrosis infections and the evolution of antibiotic resistance

Authors: Ryan A. Bronson, BS[1], Chhavi Gupta, PhD[2], Abigail L. Manson, PhD[1], Jan A. Nguyen[2], MS, Asli Bahadirli-Talbott, MS[2], Nicole M. Parrish, PhD[3], Ashlee M. Earl, PhD[1]* and Keira A. Cohen, MD[2]*

Affiliations:
[1]Infectious Disease and Microbiome Program, Broad Institute of MIT and Harvard
[2]Division of Pulmonary and Critical Care Medicine, Johns Hopkins University School of Medicine
[3]Department of Pathology, Johns Hopkins University School of Medicine
*These last authors contributed equally.

Corresponding Author:
Keira. A. Cohen, MD
Assistant Professor of Medicine
Division of Pulmonary and Critical Care Medicine
Johns Hopkins University School of Medicine
1830 E. Monument St., 5th Floor
Baltimore, MD, 21287
E-mail: kcohen8@jhmi.edu

Supplementary Note 1

Known drug resistance mechanisms explain the majority of phenotypic resistance to clarithromycin and amikacin

In order to validate use of phenotype/genotype investigations of resistance in these subspecies, we first performed phenotypic drug susceptibility testing (DST) by minimum inhibitory concentration (MIC) determination for clarithromycin and amikacin for the 30 MAB clinical isolates that were newly sequenced for this study (**Methods**). Among the 30 phenotyped strains with high quality genomic data, we observed higher clarithromycin MICs among the 20 *M. abscessus* isolates relative to the ten *M. massiliense*, as expected (**Supplementary Figures 5a-b**). On day 3 of clarithromycin MIC testing, only 60% (12/20) of M. abscessus isolates had high-level phenotypic resistance (MIC ≥ 8μg/mL) to clarithromycin, but this increased to 90% (18/20) on day 14, indicative of inducible macrolide resistance 26. For *M. massiliense*, no isolates were found to have high-level clarithromycin resistance on day 3, however, 3/10 isolates had an MIC = 8μg/mL on day 14.

Most observed resistance to clarithromycin could be explained by known genetic determinants (**Methods; Supplementary Table 4**), with overall sensitivity and specificity for prediction of clarithromycin resistance of 80.1% and 100%, respectively. For these isolates, constitutive resistance to clarithromycin in MAB was attributed to mutations in the 23S rRNA gene *rrl* [23,25], whereas inducible resistance was mediated by *erm*(41) [26] (**Table 1**).

Only a single isolate of *M. abscessus* contained a resistance mutation in *rrl* with the canonical A2271G (A2059G in *E. coli* numbering) variant [23,25], and had a corresponding high-level of resistance to clarithromycin (MIC > 16μg/mL) (**Supplementary Figures 5c-d**). As expected, 100% (17/17) of *M. abscessus* with a wildtype (T28) *erm*(41) exhibited high-level phenotypic resistance to clarithromycin. The remaining three *M. abscessus* isolates contained a T28C *erm*(41) variant [26,27], which renders *erm*(41) non-functional, and confers susceptibility to macrolides. Two of these three isolates had lower MICs to clarithromycin, whereas one isolate was phenotypically resistant, with an MIC of 16μg/mL, suggesting involvement of alternate resistance mechanism for clarithromycin, as has been suggested by other studies [21].

For *M. massiliense*, a known 276-bp deletion in *erm*(41), which has been shown to confer susceptibility to clarithromycin [26–28], was identified in all *M. massiliense* isolates. All 10 of the *M. massiliense* isolates in our dataset had lower day 14 MICs to clarithromycin, when compared to *M. abscessus* with WT *erm*. We did not observe any *rrl* variants in this subspecies.

With respect to amikacin resistance, five (16.6%) of the 30 isolates exhibited phenotypic resistance to amikacin (MIC ≥ 64μg/mL) (**Supplementary Figures 5e-f**). The overall sensitivity and specificity for prediction of amikacin resistance based on known genetic features were 80% and 100%, respectively. The five resistant isolates were all *M. abscessus* and represent specimens from three different patients (three isolates from a single patient, and two isolates from two different patients; together, 11% of patients). The three resistant isolates from the same individual, and an isolate from a second individual each contained the previously identified canonical *rrs* A1374G (A1408G in *E. coli* nomenclature) variant that confers high-level amikacin resistance [24]. However, a resistant isolate from the third person—which had MIC testing performed a total of five times to confirm phenotype—did not contain any known resistance markers for amikacin (**Supplementary Table 5**), suggesting that alternative resistance mechanisms to amikacin remain undiscovered.

Supplementary Figures

Supplementary Figure 1. *M. bolletii* isolates in our dataset were only sampled from cystic fibrosis patients. Single-copy core phylogenetic trees of 30 isolates of *M. bolletii*, all from CF patients from Bryant et al [14]. **a)** Country of origin for each isolate. **b)** Drug resistance predictions. **c)** Clusters of isolates with core SNP distance < 500 are indicated with alternating grayscale arcs on the circle (**Methods**). No cluster included sufficient members to be considered dominant circulating clones based on our definition.

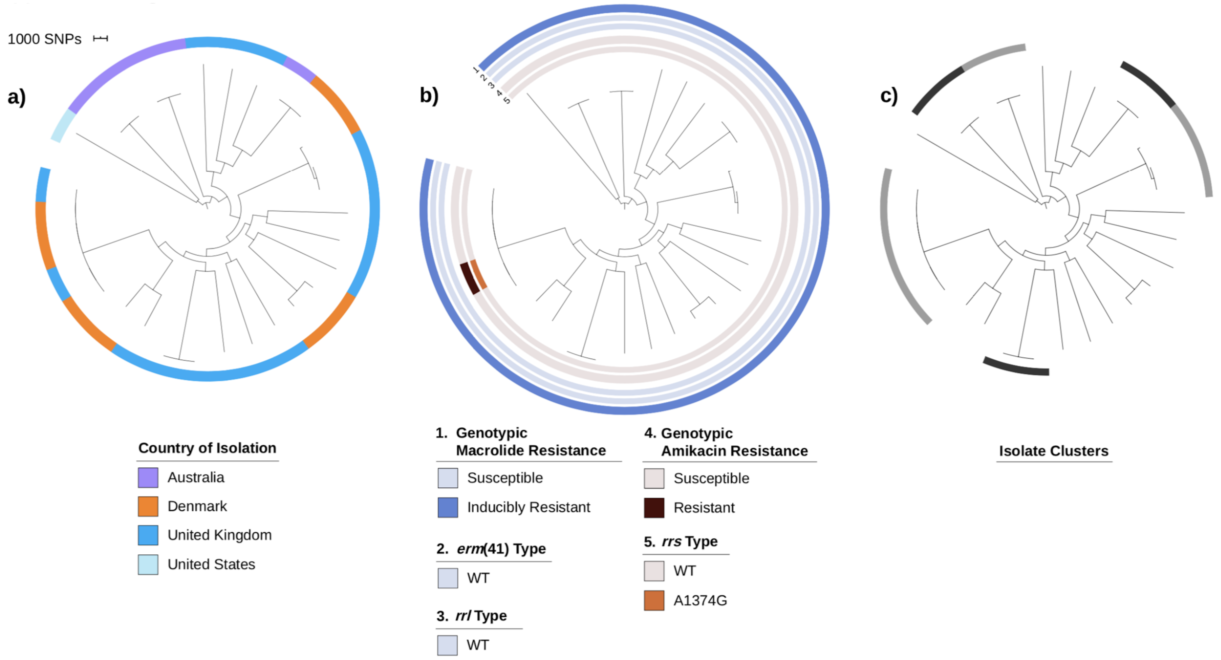

Supplementary Figure 2. Clustering by core SNPs revealed extensive clustering and global, dominant circulating clones. Clusters, calculated using hierarchical clustering with a 500 core SNP distance threshold (Methods), are indicated with alternating grayscale arcs on the circle for **a)** *M. massiliense* and **b)** *M. abscessus*. Isolates connected with a grey arc correspond to those falling within the 500 SNP threshold. DCCs, containing at least 10 isolates from more than one country, are labeled and numbered in descending order of size. DCCs A1, A2 and M1 corresponded to *M. abscessus* clusters 1 and 2, and *M. massiliense* cluster 1, respectively, from Bryant et al.

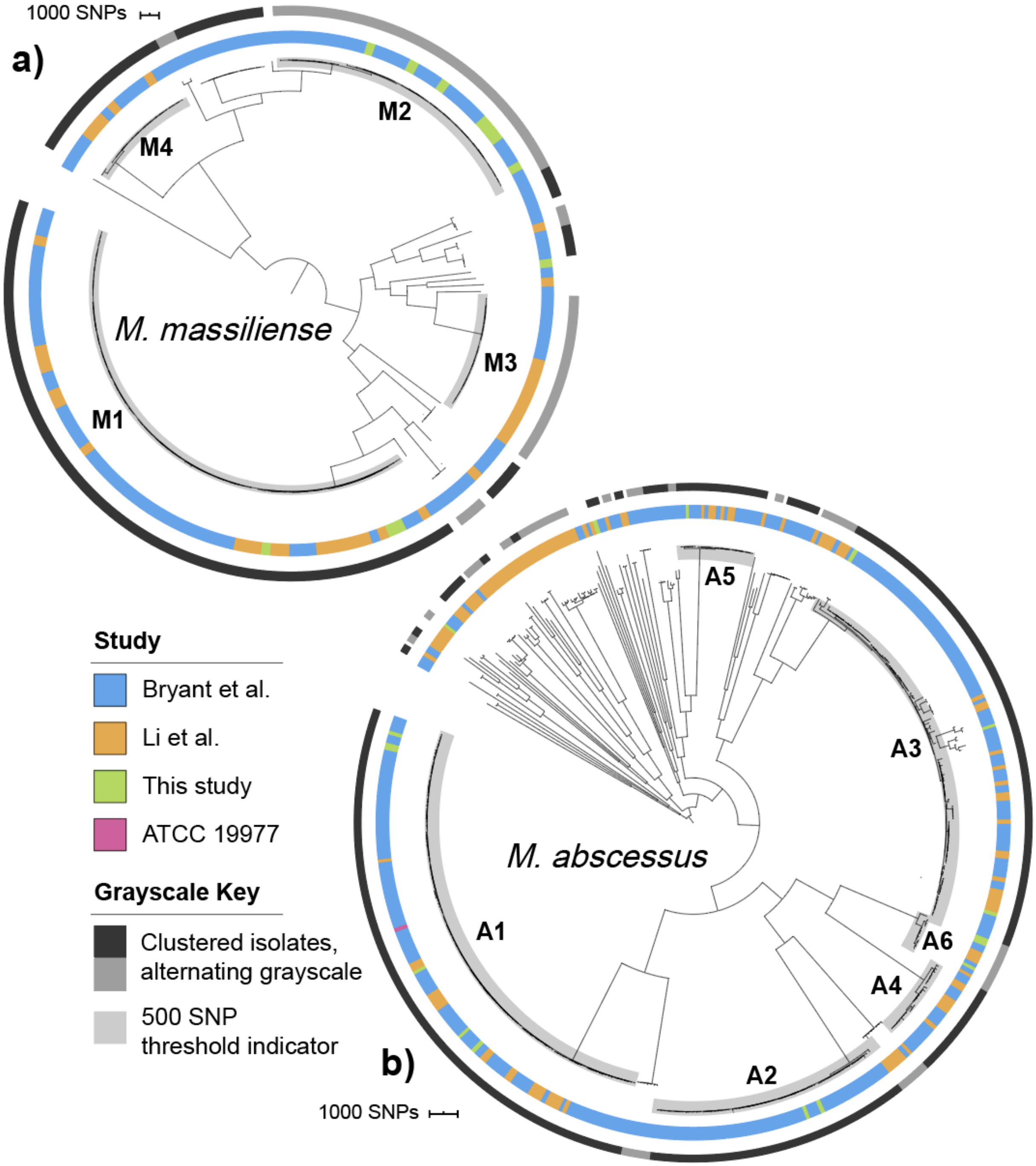

Supplementary Figure 3. Individual phylogenies for each DCC confirm nesting of CF and non-CF isolates. These higher resolution trees were constructed using the same methods as the subspecies-specific trees. Country of isolation and host CF-status are shown for each isolate.

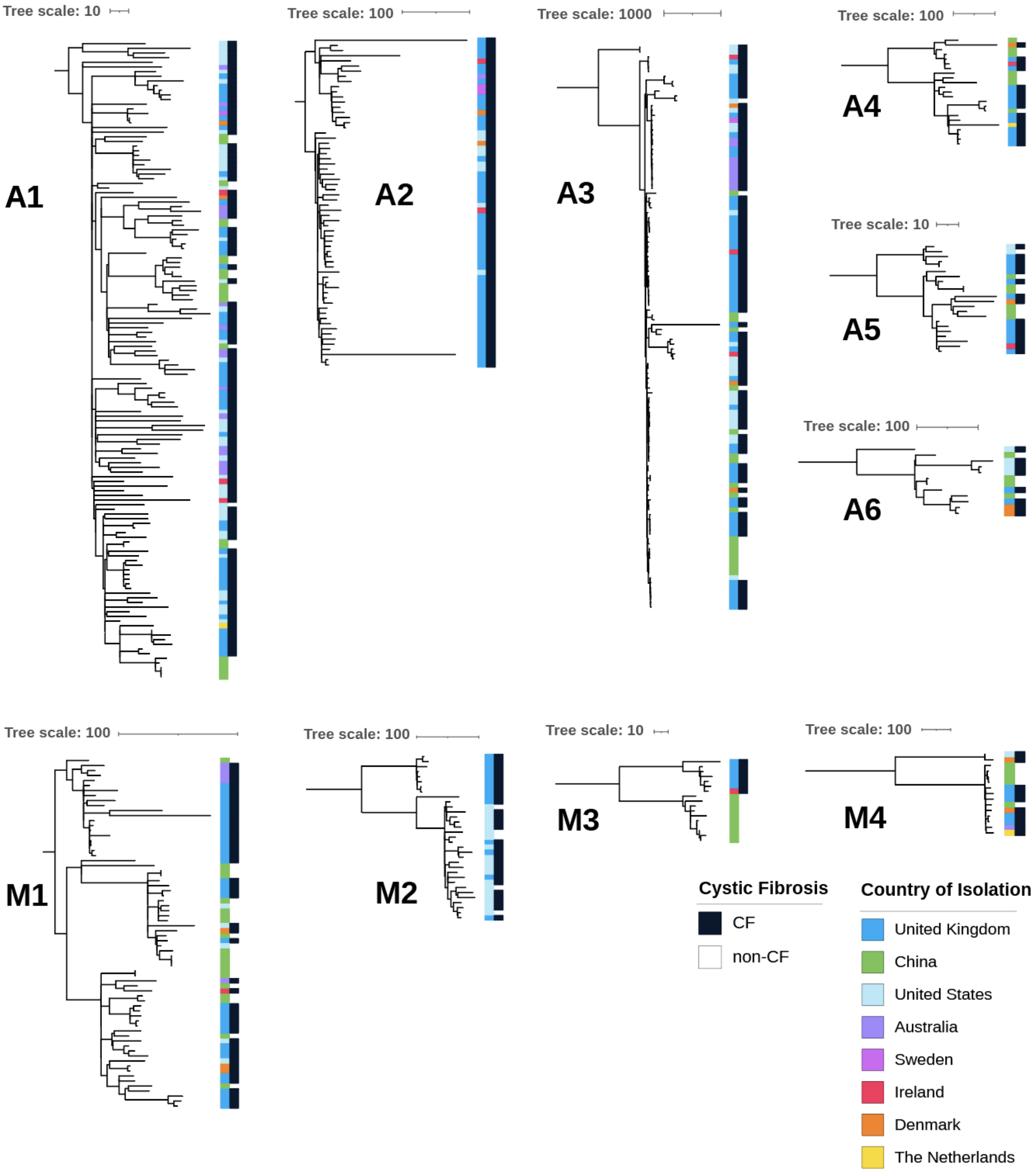

Supplementary Figure 4. Pairwise SNPs distinguished within-patient isolate pairs from between-patient isolate pairs. Precision and recall were calculated for different pairwise SNP thresholds (blue dots) for classification of within-patient isolate pairs in **a)** *M. abscessus* and **b)** *M. massiliense*. The no skill performance (the performance of a random classifier, which corresponds to the fraction of true positive samples in the dataset) is indicated by the yellow dashed lines. Thresholds for every increment of 5 are indicated in black. We chose the threshold that maximized F1 score (20 SNPs for *M. abscessus* and 15 SNPs for *M. massiliense*, corresponding to F1 scores of 0.87 and 0.72, respectively).

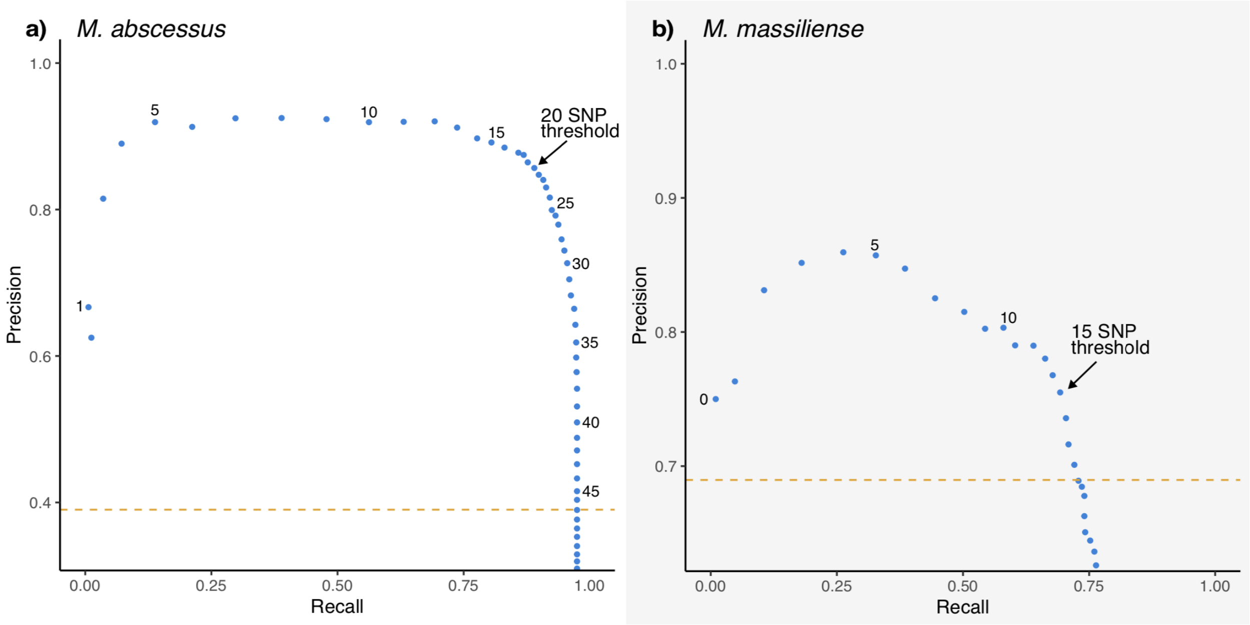

Supplementary Figure 5. MIC determination for clarithromycin and amikacin. A total of 30 newly sequenced clinical isolates of MAB (20 *M. abscessus* and 10 *M. massiliense*) underwent MIC determination to clarithromycin and amikacin. **a)** day 3 drug susceptibility for clarithromycin by MAB subspecies, **b)** day 14 drug susceptibility for clarithromycin by MAB subspecies, **c)** day 3 drug susceptibility for clarithromycin by genotype, **d)** day 3 drug susceptibility for clarithromycin by genotype, **e)** day 3 drug susceptibility for amikacin by MAB subspecies, **f)** day 3 drug susceptibility for amikacin by genotype.

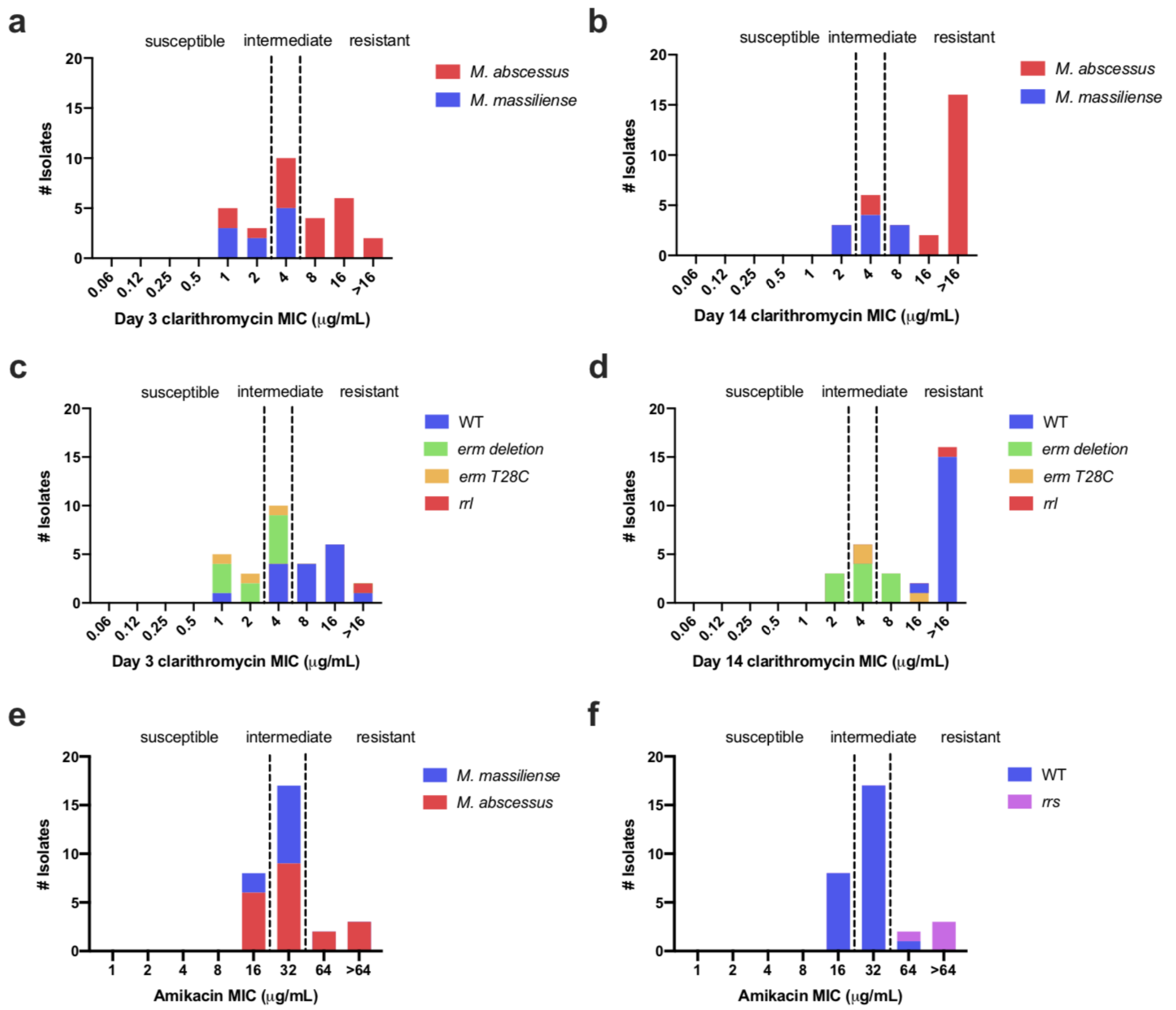

Supplementary Tables

Supplementary Table 1. Datasets included, by isolate and patient.

a)	**Number of isolates by study** [*]					
study	*M. abscessus*	*M. massiliense*	*M. bolletii*	mixed**	Total number of isolates represented	Total number of patients represented
Newly sequenced	20	10	0	0	30	27
Bryant	736	256	91	3	1086	500
Li	124	38	0	0	162	162
Total	880	304	91	3	1278	689
**b)**	**Number of unique patient isolates, excluding low-quality assemblies** [***]					
study	*M. abscessus*	*M. massiliense*	*M. bolletii*	mixed	Total number of isolates represented	Total number of patients represented
Newly sequenced	17	10	0	0	27	27
Bryant	356	123	30	0	509	498
Li	121	38	0	0	159	159
Total	494	171	30	0	695	684

[*] We selected one isolate per subspecies per patient from each longitudinal series. Thus, for the Bryant subset, the number of strains can exceed the number of patients

[**] Samples identified as mixed by Bryant et al. are indicated here. Samples identified as mixed from among the newly sequenced isolates were removed from all analyses and are not listed in this table (see Methods).

[***] All assemblies with greater than 100 scaffolds were removed from further analysis

Supplementary Table 2. Newly sequenced MAB isolates with sample identification, BioProject and accession numbers, subspecies, host CF status, median MICs for amikacin and clarithromycin (day 3 and day 14), and presence of drug resistance mutations.

Sample Name	BioProject	Accession	Subspecies	Host CF-status	Amikacin MIC	Clari MIC (d 3)	Clari MIC (d 14)	*erm*	*rrl*	*rrs*
JHN_AB_0001_1A	PRJNA523365	SAMN11100994	abscessus	non-CF	32	8	>16			
JHN_AB_0002_1	PRJNA523365	SAMN11100987	massiliense	non-CF	16	1	1.5	deletion		
JHN_AB_0003_1	PRJNA523365	SAMN11100988	abscessus	CF	16	>16	>16		A2271N	
JHN_AB_0004_1B[1]	PRJNA523365	SAMN11100989	abscessus	CF	16	5	>16			
JHN_AB_0004_2	PRJNA523365	SAMN11100991	abscessus	CF	32	8	>16			
JHN_AB_0005_2	PRJNA523365	SAMN11100995	abscessus	CF	64	8	>16			A1374N
JHN_AB_0006_2	PRJNA523365	SAMN11101019	abscessus	CF	>64	16	>16			A1374N
JHN_AB_0006_3	PRJNA523365	SAMN11101018	abscessus	CF	>64	16	>16			A1374N
JHN_AB_0006_4	PRJNA523365	SAMN11100998	abscessus	CF	>64	16	>16			A1374N
JHN_AB_0008_1	PRJNA523365	SAMN11101013	abscessus	CF	32	4	>16			
JHN_AB_0009_1	PRJNA523365	SAMN11100992	abscessus	non-CF	32	4	>16			
JHN_AB_0010_1	PRJNA523365	SAMN11100993	abscessus	non-CF	24	1.5	5	T28C		
JHN_AB_0013_1	PRJNA523365	SAMN11100996	massiliense	CF	24	1.5	2	deletion		
JHN_AB_0016_1	PRJNA523365	SAMN11100999	abscessus	CF	24	8	>16			
JHN_AB_0017_1	PRJNA523365	SAMN11101000	abscessus	non-CF	16	1.25	>16			
JHN_AB_0018_1	PRJNA523365	SAMN11101001	abscessus	CF	64	16	>16			
JHN_AB_0019_1	PRJNA523365	SAMN11101002	massiliense	non-CF	32	3	8	deletion		
JHN_AB_0020_1	PRJNA523365	SAMN11101003	massiliense	non-CF	32	4	6	deletion		
JHN_AB_0021_1	PRJNA523365	SAMN11101004	abscessus	CF	32	3	16	T28C		
JHN_AB_0022_1	PRJNA523365	SAMN11101005	massiliense	CF	30	1.25	2	deletion		
JHN_AB_0023_1	PRJNA523365	SAMN11101006	massiliense	non-CF	32	1.5	8	deletion		
JHN_AB_0024_1	PRJNA523365	SAMN11101007	massiliense	non-CF	32	1	4	deletion		
JHN_AB_0025_1	PRJNA523365	SAMN11101008	abscessus	CF	32	>16	>16			
JHN_AB_0027_1	PRJNA523365	SAMN11101010	abscessus	CF	16	1	4	T28C		
JHN_AB_0028_1	PRJNA523365	SAMN11101011	massiliense	non-CF	32	4	10	deletion		
JHN_AB_0031_1	PRJNA523365	SAMN11101014	abscessus	CF	48	6	16			
JHN_AB_0032_1	PRJNA523365	SAMN11101015	massiliense	non-CF	32	4	4	deletion		
JHN_AB_0033_1	PRJNA523365	SAMN11101016	massiliense	non-CF	16	4	4	deletion		
JHN_AB_0034_1	PRJNA523365	SAMN11101017	abscessus	non-CF	16	16	>16			
JHN_AB_0037_1	PRJNA523365	SAMN11101020	abscessus	CF	16	12	>16			
JHN_AB_19977	PRJNA523365	SAMN11100986	abscessus	non-CF						

[1]Shaded rows indicate isolates that derived from the same person at different timepoints.

Supplementary Table 3. Dominant circulating clones were globally distributed.

Clone[1]	Subspecies	Count[2]	SNP range[3]	Countries where isolates originated
A1	*abscessus*	137	0-94	Australia, China, Denmark, Ireland, Netherlands, Sweden, UK, US
A3	*abscessus*	116	0-219	Australia, China, Denmark, Ireland, Sweden, UK, US
A2	*abscessus*	64	8-85	Australia, Denmark, Ireland, Sweden, UK, US
A4	*abscessus*	23	8-145	China, Denmark, Ireland, Netherlands, UK, US
A5	*abscessus*	22	1-50	China, Denmark, Ireland, UK, US
A6	*abscessus*	11	3-95	China, Denmark, UK, US
M1	*massiliense*	70	0-60	Australia, China, Denmark, Ireland, UK, US
M2	*massiliense*	33	3-55	UK, US
M3	*massiliense*	17	0-43	China, Ireland, UK
M4	*massiliense*	15	1-67	Australia, China, Denmark, Netherlands, UK, US

[1]DCC (**Figure 1**)

[2]Number of isolates within the DCC

[3]Range of SNPs between pairwise combinations of isolates within this DCC

Supplementary Table 4. All closely-related pairs of MAB isolates that were separated by fewer pairwise SNPs than our subspecies-specific SNP thresholds and were unlikely to be epidemiologically linked (different CF-status and/or country of isolation).

Isolate 1[1]	Isolate 2[1]	Subspecies	Pairwise SNPs[2]	Isolate 1 CF-status[3]	Isolate 2 CF-status	Isolate 1 country[4]	Isolate 2 country	DCC[3]
10702_1_3	10250_1_13	*abscessus*	6	CF	CF	Denmark	United Kingdom	A1
10702_1_3	10665_4_36	*abscessus*	9	CF	CF	Denmark	United Kingdom	A1
12163_2_18	10465_1_61	*abscessus*	9	CF	CF	United Kingdom	United States	A1
12163_2_16	ASM280061v1	*abscessus*	10	CF	non-CF	United Kingdom	China	A4
12163_2_51	10465_1_61	*abscessus*	10	CF	CF	United Kingdom	United States	A1
11893_6_53	10665_4_49	*abscessus*	13	CF	CF	Ireland	United Kingdom	A5
JHN_AB_0017_1	10660_1_17	*abscessus*	13	non-CF	CF	United States	United States	A5
10208_3_59	ASM280068v1	*abscessus*	13	CF	non-CF	United Kingdom	China	A4
10660_1_6	10660_1_22	*abscessus*	13	CF	CF	United States	United Kingdom	A1
12163_2_56	11893_6_53	*abscessus*	14	CF	CF	United Kingdom	Ireland	A5
ASM280081v1	10208_3_63	*abscessus*	14	non-CF	CF	China	United Kingdom	A5
ASM280096v1	10208_3_63	*abscessus*	14	non-CF	CF	China	United Kingdom	A5
12163_2_1	JHN_AB_0017_1	*abscessus*	14	CF	non-CF	United Kingdom	United States	A5
12163_2_2	JHN_AB_0017_1	*abscessus*	14	CF	non-CF	United Kingdom	United States	A5
10465_1_59	10660_1_22	*abscessus*	14	CF	CF	United States	United Kingdom	A1
12163_2_47	ASM280046v1	*abscessus*	14	CF	non-CF	United Kingdom	China	A1
10208_3_63	ASM280084v1	*abscessus*	15	CF	non-CF	United Kingdom	China	A5
10665_4_36	12082_5_83	*abscessus*	15	CF	CF	United Kingdom	Sweden	A1
12163_2_2	10660_1_17	*abscessus*	16	CF	CF	United Kingdom	United States	A5
10208_3_59	10625_5_4	*abscessus*	16	CF	CF	United Kingdom	Ireland	A4
10702_1_3	12082_5_83	*abscessus*	16	CF	CF	Denmark	Sweden	A1
10702_1_86	10660_1_22	*abscessus*	16	CF	CF	United States	United Kingdom	A1
12163_2_1	10660_1_17	*abscessus*	17	CF	CF	United Kingdom	United States	A5
10702_1_26	10660_1_22	*abscessus*	17	CF	CF	United States	United Kingdom	A1
11893_6_53	12163_1_95	*abscessus*	18	CF	CF	Ireland	United Kingdom	A5
10208_3_61	11893_6_53	*abscessus*	19	CF	CF	United Kingdom	Ireland	A5
11893_6_53	12082_5_69	*abscessus*	19	CF	CF	Ireland	United Kingdom	A5
10625_4_20	JHN_AB_0017_1	*abscessus*	19	CF	non-CF	United Kingdom	United States	A5
12163_2_47	ASM280070v1	*abscessus*	19	CF	non-CF	United Kingdom	China	A1
12163_2_47	ASM280062v1	*abscessus*	19	CF	non-CF	United Kingdom	China	A1
12082_5_80	ASM280204v1	*abscessus*	20	CF	non-CF	United Kingdom	China	A1
10660_1_35	10660_1_22	*abscessus*	20	CF	CF	United States	United Kingdom	A1
10660_1_13	10660_1_22	*abscessus*	20	CF	CF	United States	United Kingdom	A1
10625_4_8	10465_1_55	*abscessus*	20	CF	CF	United Kingdom	United States	A1
10465_1_61	12082_5_68	*abscessus*	20	CF	CF	United States	United Kingdom	A1

10625_5_30	10625_5_8	*massiliense*	7	CF	CF	United Kingdom	Ireland	M3
JHN_AB_0024_1	ASM280002v1	*massiliense*	9	non-CF	non-CF	United States	China	M1
JHN_AB_0002_1	10208_3_60	*massiliense*	12	non-CF	CF	United States	United Kingdom	M2
10625_5_8	10208_3_3	*massiliense*	13	CF	CF	Ireland	United Kingdom	M3
JHN_AB_0002_1	10702_1_48	*massiliense*	13	non-CF	CF	United States	United States	M2
JHN_AB_0023_1	ASM280006v1	*massiliense*	13	non-CF	non-CF	United States	China	M1
JHN_AB_0024_1	10250_1_76	*massiliense*	14	non-CF	CF	United States	United Kingdom	M1
JHN_AB_0023_1	ASM280182v1	*massiliense*	14	non-CF	non-CF	United States	China	M1
10208_3_58	10625_5_8	*massiliense*	15	CF	CF	United Kingdom	Ireland	M3
10625_5_8	10625_5_27	*massiliense*	15	CF	CF	Ireland	United Kingdom	M3
JHN_AB_0023_1	ASM279965v1	*massiliense*	15	non-CF	non-CF	United States	China	M1

[1]Identifiers listed correspond to either an assembly accession ("ASM" in isolates from Li et al [17]), isolate codes from this study ("JHN"), or the isolate codes from Bryant et al [14].
[2]Number of SNPs different in all pairwise alignments of shared orthogroups after removing potential recombination (**Methods**). All pairs below the subspecies-specific SNP thresholds (20 SNPs for *M. abscessus* and below 15 SNPs for *M. massiliense*) are listed here.
[3]Unshaded rows represent isolate pairs that differed by country of isolation.
[4]Shaded rows represent isolate pairs that differed by CF-status as well as country of isolation (with the exception of two rows that only differed by CF-status).
[5]DCCs as listed in **Supplementary Figure 2**

Supplementary Table 5. Genotypic resistance definitions for clarithromycin and amikacin.

**Drug**	**Phenotype**	**MAB numbering**	***E. coli* numbering**
Clarithromycin	Constitutive resistance	*rrl* T371C<br>*rrl* G795A<br>*rrl* A1932G<br>*rrl* A2269G<br>***r**rl* A2270N<br>*rrl* A2271N<br>*rrl* G2281A<br>*rrl* A2293C	*rrl* A2057N<br>*rrl* A2058N<br>*rrl* A2059N<br>*rrl* G2069A<br>*rrl* A2082C
	Inducible resistance	WT *erm* at T28	
	Susceptible	*erm* T28C<br>*erm* deletion	
Amikacin	Constitutive resistance	*rrs* A1374G	*rrs* T1406A<br>*rrs* A1408G<br>*rrs* C1409T

Supplementary Table 6. AMR markers and phenotypic prediction percent by isolate CF-status.

	**% of cystic fibrosis isolates**	**% of non-cystic fibrosis isolates**	**Are CF and non-CF significantly different?***
Genotype			
rrl variant	7.5%	6.9%	1
rrl WT	92.5%	93.1%	
erm(41) deletion	23.8%	26.6%	0.4758
erm(41) non-truncated	76.2%	73.4%	
erm(41) C28	15%	9.8%	0.09744
erm(41) T28	85%	90.2%	
rrs variant	5.6%	99.4%	0.003858**
rrs WT	94.4%	0.6%	
Predicted phenotype			
macrolide constitutive resistance	7.5%	6.9%	0.7847
macrolide inducible resistance	58.2%	61.3%	
macrolide susceptible	34.4%	31.8%	
amikacin resistant	5.6%	99.4%	0.003858**
amikacin susceptible	94.4%	0.6%	

*Significance level comparing CF to non-CF for each row was performed using a two-sided Fisher's exact test. ** = $p \leq 0.01$

Supplementary Table 7. rRNA SNP frequencies by subspecies.

	Counts (Frequencies)		
**SNP**	***M. abscessus***	***M. massiliense***	***M. bolletii***
rrl G795A	5 (1.0%)	0	0
rrl A2270N	13 (2.6%)	22 (12.9%)	0
rrl A2271N	4 (0.8%)	4 (2.3%)	0
rrl G2281A	2 (0.4%)	0	0
rrl A2293T	1 (0.1%)	0	0
rrs A1374G	20 (4.0%)	9 (5.3%)	1 (3.3%)

Supplementary Table 8. GenBank assembly references used for MAB subspecies identification.

GenBank assembly accession	MAB subspecies
GCA_000069185.1	*M. abscessus*
GCA_001606295.1	*M. abscessus*
GCA_001606315.1	*M. abscessus*
GCA_001606335.1	*M. abscessus*
GCA_001610655.1	*M. abscessus*
GCA_001677155.1	*M. abscessus*
GCA_001677195.1	*M. abscessus*
GCA_000445035.1	*M. bolletii*
GCA_001606195.1	*M. bolletii*
GCA_003609715.1	*M. bolletii*
GCA_000277775.2	*M. massiliense*
GCA_000497265.2	*M. massiliense*
GCA_001606215.1	*M. massiliense*
GCA_001606235.1	*M. massiliense*
GCA_001606255.1	*M. massiliense*
GCA_001606275.1	*M. massiliense*
GCA_001610615.1	*M. massiliense*
GCA_001610635.1	*M. massiliense*
GCA_001610675.1	*M. massiliense*
GCA_001677095.1	*M. massiliense*
GCA_001677175.1	*M. massiliense*
GCA_001677215.1	*M. massiliense*
GCA_002140035.1	*M. massiliense*
GCA_004209815.1	*M. massiliense*
GCA_004209835.1	*M. massiliense*
