# Supplementary material for: Global phylogenomic analyses of Mycobacterium abscessus provide context for non cystic fibrosis infections and the evolution of antibiotic resistance
Source: Nat Commun. 2021 Aug 26;12:5145. doi: 10.1038/s41467-021-25484-9 (PMC8390669; doi:10.1038/s41467-021-25484-9)
Supplement: Supplementary file 2 — Reporting Summary [file 41467_2021_25484_MOESM2_ESM.pdf]

Corresponding author(s): Keira A. Cohen, MD

Last updated by author(s): Aug 11, 2021

Reporting Summary

Nature Research wishes to improve the reproducibility of the work that we publish. This form provides structure for consistency and transparency in reporting. For further information on Nature Research policies, see our Editorial Policies and the Editorial Policy Checklist.

Statistics

For all statistical analyses, confirm that the following items are present in the figure legend, table legend, main text, or Methods section.

n/a	Confirmed	
☐	☒	The exact sample size (n) for each experimental group/condition, given as a discrete number and unit of measurement
☐	☒	A statement on whether measurements were taken from distinct samples or whether the same sample was measured repeatedly
☐	☒	The statistical test(s) used AND whether they are one- or two-sided<br>*Only common tests should be described solely by name; describe more complex techniques in the Methods section.*
☒	☐	A description of all covariates tested
☐	☒	A description of any assumptions or corrections, such as tests of normality and adjustment for multiple comparisons
☒	☐	A full description of the statistical parameters including central tendency (e.g. means) or other basic estimates (e.g. regression coefficient) AND variation (e.g. standard deviation) or associated estimates of uncertainty (e.g. confidence intervals)
☐	☒	For null hypothesis testing, the test statistic (e.g. F, t, r) with confidence intervals, effect sizes, degrees of freedom and P value noted<br>*Give P values as exact values whenever suitable.*
☒	☐	For Bayesian analysis, information on the choice of priors and Markov chain Monte Carlo settings
☒	☐	For hierarchical and complex designs, identification of the appropriate level for tests and full reporting of outcomes
☒	☐	Estimates of effect sizes (e.g. Cohen's d, Pearson's r), indicating how they were calculated

Our web collection on statistics for biologists contains articles on many of the points above.

Software and code

Policy information about availability of computer code

Data collection	No software was used to collect data for this study.
Data analysis	The software used in this study was standard for the field and usage was described in Methods: we used parsnp (v1.2), Gubbins(v2.3.1), RAxML (v7.3.3), SINA from Silva (v1.2.11), BLAST (v2.2.30), Muscle (v3.8.31), Deepbinner (v0.2.0), Porechop (v0.2.3), Trim Galore (v0.5.0), Unicycler (v0.4.4), bwa mem (v0.7.17), Pilon (v1.23), GAEMR (v1.0.1), Vesper (Prodigal, tRNAscan-SE, RNAmmer) (v1.0), SynerClust (version January 10, 2020), and SciPy (v1.1.0). Any other scripts used were small custom scripts also described in Methods.

For manuscripts utilizing custom algorithms or software that are central to the research but not yet described in published literature, software must be made available to editors and reviewers. We strongly encourage code deposition in a community repository (e.g. GitHub). See the Nature Research guidelines for submitting code & software for further information.

Data

Policy information about availability of data

All manuscripts must include a data availability statement. This statement should provide the following information, where applicable:

- Accession codes, unique identifiers, or web links for publicly available datasets
- A list of figures that have associated raw data
- A description of any restrictions on data availability

The genomic data generated in this study have been deposited in NCBI SRA under Bioproject PRJNA 523365 [https://www.ncbi.nlm.nih.gov/bioproject/PRJNA523365]. The accession codes and phenotypic data generated in this study are provided in the Supplementary Table 2. Publicly available genome sequences utilized in this project include NCBI under BioProject PRJNA 398137 [https://www.ncbi.nlm.nih.gov/bioproject/PRJNA398137] and the European Nucleotide Archive under project accession PRJEB2779 [https://www.ebi.ac.uk/ena/browser/view/PRJEB2779]. GenBank assembly references used for MAB subspecies identification are found in Table S8.

Field-specific reporting

Please select the one below that is the best fit for your research. If you are not sure, read the appropriate sections before making your selection.

- [x] Life sciences
- [ ] Behavioural & social sciences
- [ ] Ecological, evolutionary & environmental sciences

For a reference copy of the document with all sections, see nature.com/documents/nr-reporting-summary-flat.pdf

Life sciences study design

All studies must disclose on these points even when the disclosure is negative.

Sample size	We combined the largest previously published genomic studies of M. abscessus of CF and non-CF individuals. Newly sequenced isolates were first identified by distinct host features to ensure representation from individuals both with and without cystic fibrosis. Inclusion was limited to only a single isolate per patient, rather than serial isolates, unless the serial isolates displayed differing drug susceptibility patterns. Isolates of diverse drug susceptibility patterns from both CF and non-CF hosts were randomly selected for inclusion. A sample size calculation was not performed, however, the current sampling strategy was sufficient to identify nesting of CF and non-CF isolates within DCCs on the phylogeny.
Data exclusions	Using pre-established quality criteria described in the methods section, we excluded low-quality assemblies from all subsequent analyses. Numbers of low quality samples that were excluded are listed in Table S1, and these excluded samples were not used for any genomic analyses.
Replication	Drug susceptibility phenotyping was performed in duplicate and repeated a third time or more if there were significant differences in phenotype observed (ex: by more than one dilution on MIC testing). All attempts at experimental replication were successful.
Randomization	Randomization was not performed as there was no intervention applied.
Blinding	Genomic analysis was initially performed without knowledge of CF status or drug susceptibility pattern.

Reporting for specific materials, systems and methods

We require information from authors about some types of materials, experimental systems and methods used in many studies. Here, indicate whether each material, system or method listed is relevant to your study. If you are not sure if a list item applies to your research, read the appropriate section before selecting a response.

Materials & experimental systems

n/a	Involved in the study	
x		Antibodies
x		Eukaryotic cell lines
x		Palaeontology and archaeology
x		Animals and other organisms
	x	Human research participants
x		Clinical data
x		Dual use research of concern

Methods

n/a	Involved in the study	
x		ChIP-seq
x		Flow cytometry
x		MRI-based neuroimaging

Human research participants

Policy information about studies involving human research participants

Population characteristics	Mycobacterial isolates from individuals both with cystic fibrosis and without cystic fibrosis were selected for inclusion. 53.3% of included participants were male. The average age at the time of sample collection was 41.5 years +/- 21 years.
Recruitment	M. abscessus isolates from the Johns Hopkins Clinical Mycobacteriology Laboratory were randomly chosen for inclusion.
Ethics oversight	IRB approval was obtained from the Johns Hopkins University School of Medicine IRB.

Note that full information on the approval of the study protocol must also be provided in the manuscript.
